# Study of Material Color Influences on Mechanical Characteristics of Fused Deposition Modeling Parts

**DOI:** 10.3390/ma15197039

**Published:** 2022-10-10

**Authors:** Ge Gao, Fan Xu, Jiangmin Xu, Zhenyu Liu

**Affiliations:** 1 School of Mechanical Engineering, Jiangsu University of Science and Technology, Zhenjiang 212100, China; 2 Research Institute of Marine Equipment, Jiangsu University of Science and Technology, Zhenjiang 212100, China; 3 Changchun Institute of Optics, Fine Mechanics and Physics, Chinese Academy of Sciences, Changchun 130033, China

**Keywords:** fused deposition modeling (FDM), mechanical strengths, color, ABS, PLA

## Abstract

The objective of the present work is to evaluate the influence of material color on mechanical properties of fused deposition modeling (FDM) parts. The performance of the products is evaluated by testing eight different colors of acrylonitrile butadiene styrene (ABS) and polylactic acid (PLA) material in terms of tensile strength, compressive strength, and flexural strength. The analysis of data shows a significant difference in mechanical characteristics of prints depending on filament color. For different colors, these three strengths almost follow the same rising and falling tendency. In order to explore the relationship between mechanical strengths and filament colors, the color-mixing theory and the least-squares method are adopted to fit the best ratio coefficients of different color combinations. Results are presented showing that the strength value (e.g., tensile) of the mixed color can be evaluated through that of primary colors by fitting the other strength (e.g., compressive or flexural). It is shown that the predicted value is always no more than 7% error compared with the actual strength, in spite of two-color or three-color mixtures. An additional confirmation test with seven colored PLA filaments from different suppliers was conducted to focus on the extensibility. The outcomes show the maximum fitting errors of strengths for mixed colors in all cases are within 5%, proving the effectiveness and applicability of this predicted approach. This study can bring a detailed analysis that enables better estimation of the function of material color and contributes to improving the property of FDM printed products for consumers by choosing the suitable filament color.

## 1. Introduction

Additive manufacturing (AM), also known as 3D printing, is the process of creating parts directly from three-dimensional model data by depositing materials layer by layer gradually. Compared with traditional subtractive manufacturing, AM has the following advantages such as digital production, flexibility of complex geometry, high manufacturing efficiency, resource conservation and environmental friendliness [1]. Therefore, AM plays an important role in Industry 4.0 and has the potential to be industrially revolutionary. Among many AM technologies, the most widely used nowadays is fused deposition modeling (FDM). The principle of this technique is that the thermoplastic filament is extruded from a heated nozzle, and deposited layer by layer to form the component. Due to convenient operation, rapid prototyping, minimal waste, and low cost, FDM has been experiencing a great increase in various fields, such as aerospace, biomedical, architecture, automotive, and many others in recent years [2].

FDM is a complex process with a large number of parameters that significantly impact the quality of built parts. With increasing higher demands on the structural performance of products, many researchers devoted to investigating the influence of process parameters, such as build orientation [3], raster angle [4], layer thickness [5], infill density [6], air gap [7], and others factors [8,9] to achieve desired mechanical properties. Even so, a characteristic barely evaluated is the influence of material color. In most cases, it is assumed that filament color has an insignificant relevant connection to the final quality [10]. In addition, manufacturers usually give the same characteristics for the same material regardless of the color selection. However, only a few researchers found that different colors of the same polymer resulted in obvious differences in material behavior, such as dimension tolerance [11,12], surface roughness [13], and most importantly, mechanical properties. Pandzic et al. [14] showed that color had a great influence on the elastic modulus, yield strength, tensile strength, and toughness by studying 13 different colors of PLA. Wittbrodt and Pearcec [15] experimentally presented that material color, as well as temperature, had a strong relationship with the percentage of crystallinity, and thus affected the tensile strength of PLA. The conclusions are consistent with Spina [16], whose study reported that crystallinity was color-dependent. Tymrak et al. [17] printed specimens with different colors of the same PLA, and observed notable differences in extrusion characteristics. Tanikella et al. [18] pointed out that color had a significant impact on the maximum stress that the printed component could withstand. Schwartz et al. [19] confirmed that choosing the wrong color filament for a printing task could result in a failure of a functional part if not carefully considered. By reviewing the literature, it can be noticed that all existing studies just focused on PLA filament. In fact, there are various materials on the market available for FDM users, and it is necessary to consider whether the influence of color exists for other materials and in what amount. Moreover, the internal correlation between the mechanical properties corresponding to various colors is also worthy of further investigation.

To meet this need, the present study aims to examine differences in mechanical behaviors (tensile, compressive, and flexural strength) of ABS and PLA samples with different colors, which are the most popular filament materials for FDM users. Based on the experimental data, this study brings a view of colorimetry to explain the relationship of mechanical properties between different colors. The achieved results are helpful for FDM users to select the most suitable material color for improving the structural performance of products.

## 2. Experimental Procedure

### 2.1. Materials and Machines

The ABS and PLA filaments were provided by eSUN^®^ (Beijing, China). Eight different colors (red, yellow, blue, green, black, orange, purple, and natural (translucent white)) were selected to determine the influence on the final properties of prints. All filaments were stored in a sealed package with desiccant packets to avoid moisture absorption.

**Figure 1:**
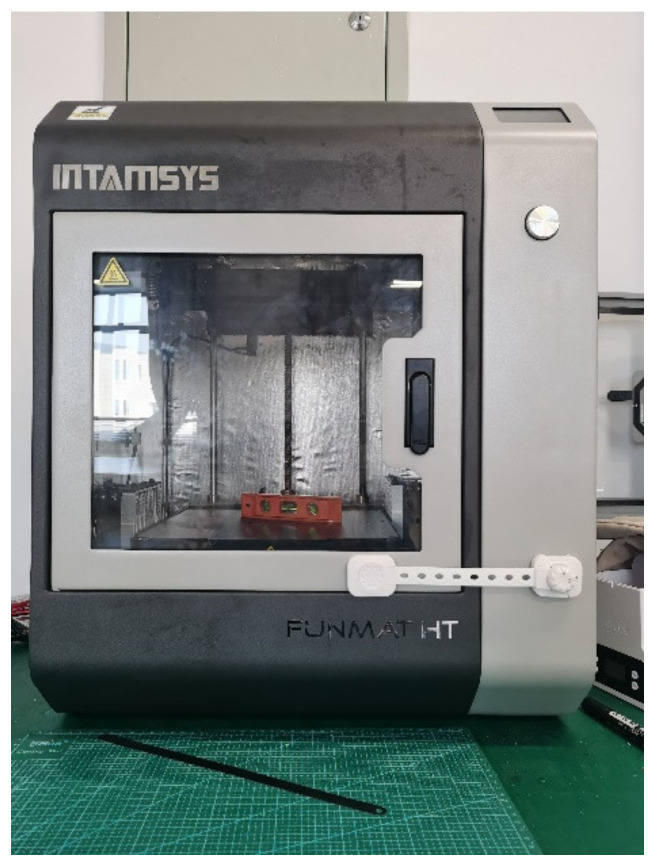
FUNMAT HT printer.

The specimens were manufactured by FUNMAT HT^®^ commercial FDM printer (Hangzhou, China), as shown in Figure 1. All specimens in different colors were printed under the same conditions, whose process parameters were included in Table 1. To reduce the influence of anisotropy of FDM parts, all specimens were produced with “flat” build orientation and 0° raster angle, which aligned the deposited filament with the direction of loading with the least number of layers.

**Figure 2:**
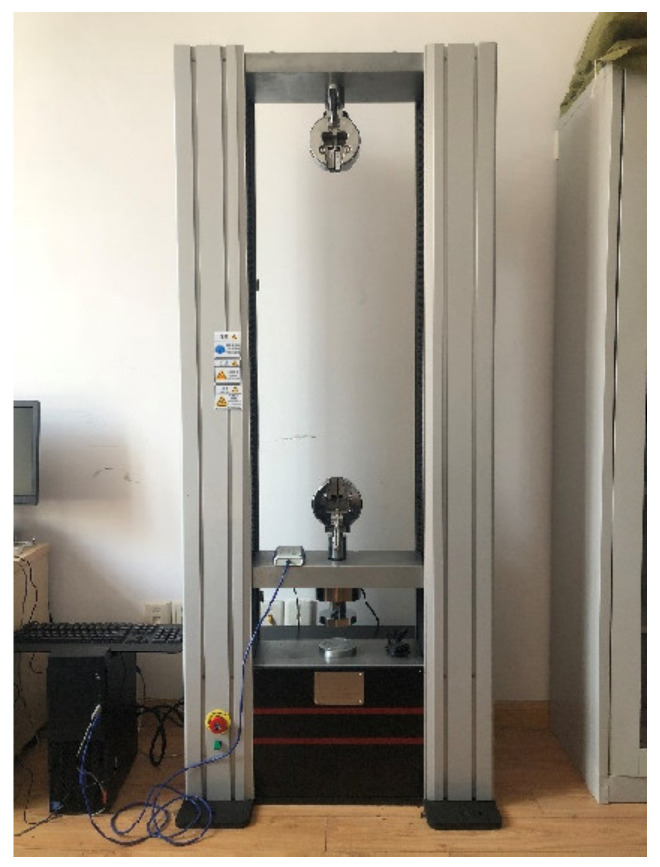
MIT-20 testing machine.

The tests were carried out using a MIT-20 electronic universal testing machine (Changzhou, China), with a measurement capacity of 20 kN, as shown in Figure 2. Due to the lack of specific testing standardization for AM processes, according to the literature review [20,21], ASTM D638 for tensile strength, ASTM D790 for flexural strength, and ASTM D695 for compressive strength were generally used, which were also adopted in this study.

### 2.2. Specimens Testing

**Figure 3:**
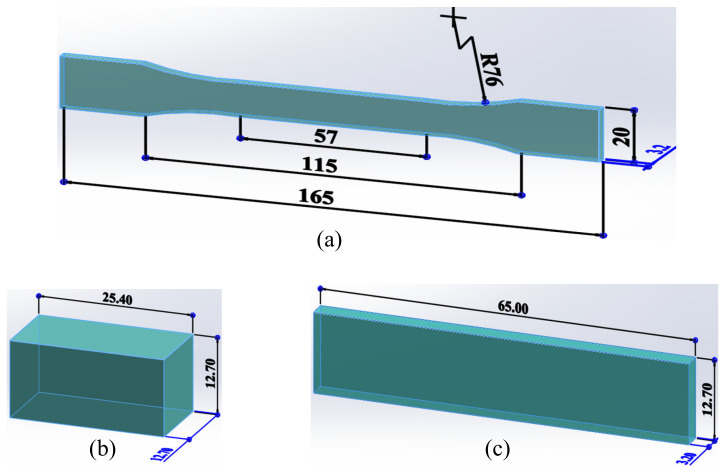
Geometry of experimental specimens: (**a**) tensile, (**b**) compressive and (**c**) flexural.

The shape and size of the tensile specimen with type I was determined according to the ASTM D638, and the test speed was 5 mm/min. The compressive specimen and flexural specimen were designed according to the aforementioned ASTM standard (Figure 3). The test speed in both cases was 2 mm/min. All specimen tests were conducted at room temperature to measure the mechanical strengths.

**Figure 4:**
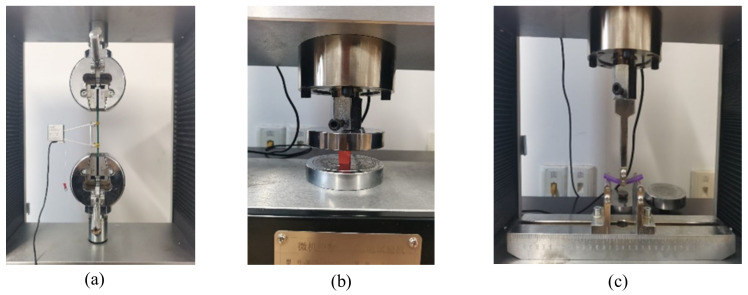
Experimental tests for specimens: (**a**) tensile, (**b**) compressive and (**c**) flexural.

**Figure 5:**
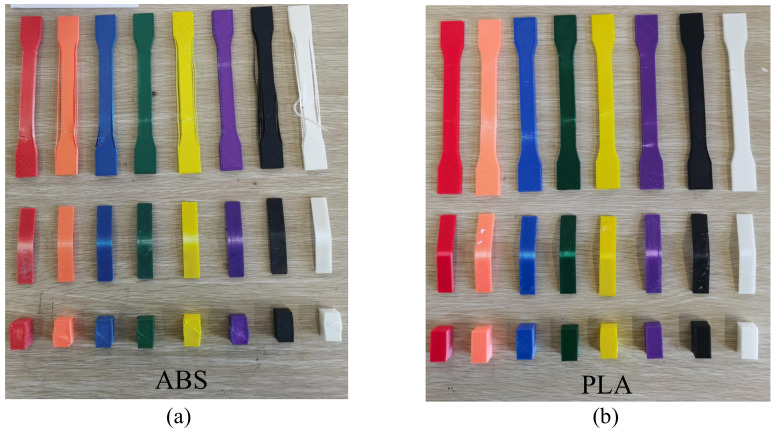
Part of broken specimens after testing: (**a**) ABS and (**b**) PLA.

In order to ensure the accuracy of the experimental result, three identical specimens for each color sample were prepared for each type of mechanical test, and the average value of data obtained was calculated as the outcome of strength (Figure 4). In total, there were seventy-two specimens corresponding to different colors and mechanical tests for ABS and PLA, respectively (Figure 5).

### 2.3. Results and Discussions

**Figure 6:**
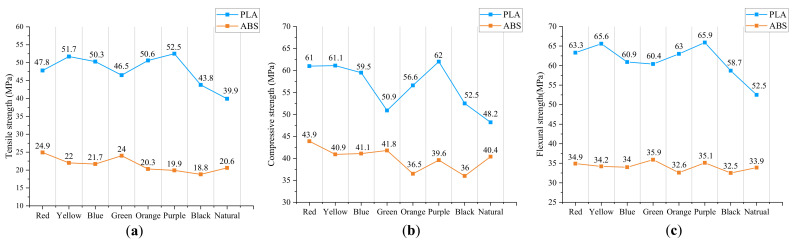
Mechanical strengths for PLA and ABS: (**a**) tensile, (**b**) compressive and (**c**) flexural.

The results of tensile strength, compressive strength, and flexural strength of ABS and PLA in all colors are plotted in Figure 6, and the numerical data is presented in Table 2. As can be seen in the data, specimens printed by PLA generally show a better performance with higher mechanical strength, compared with ABS specimens, which has been proved in previous research [22]. For PLA, tensile strength varies from 39.9 MPa to 52.5 MPa, compressive strength varies from 48.2 MPa to 62.0 MPa, and flexural strength varies from 52.5 MPa to 65.9 MPa for different colors. The maximum value of all relative differences in mechanical strengths between different colors can be up to 32% (tensile strength), and the minimum value can reach 26% (flexural strength). In addition, PLA material with purple color gives the highest values, while natural color gives the lowest values for three types of tests. In light of the values obtained, it can be demonstrated that material color has a significant influence on mechanical properties for PLA, confirming the conclusion obtained from other research [14,15].

On the other hand, in the case of ABS, tensile strength ranges from 18.8 MPa to 24.9 MPa, compressive strength ranges from 36.0 MPa to 43.9 MPa, and flexural strength ranges from 32.5 MPa to 35.9 MPa for different colors. The maximum value of all relative differences in mechanical strengths between different colors can be up to 32% (tensile strength), and the minimum value can reach 10% (flexural strength). Similar to PLA, variance in color does impact the performance of the ABS material as well. However, the effect is more pronounced on tensible and compressive strength, compared with flexural strength. Moreover, the ABS material with red color gives the highest value, and black color gives the lowest value for both tensible and compressive strength. Nevertheless, for flexural strength, the highest value and lowest value are obtained in green color and black color, respectively. The results do not seem to be in line with the previous tendency from PLA, that is, extreme values are obtained in the same colors. One possible explanation for this phenomenon is that the difference in this group of data is small, which is easily affected by various errors, e.g., manufacturing error and experimental error. In addition, the relative difference in flexural strength between red color (34.9 MPa) and green color (35.9 MPa) is only 3%. Taking into account the factor of errors, the results obtained can be acceptable.

The difference in material color is due to color additives (tint or pigment) processed into filaments. Actually, the “filler” added to the polymer can change the thermal properties, such as crystallization [15] and melt viscosity [23], which have essential effects on bond formation and adhesion between adjacent filaments, and thus mechanical properties [24,25]. It is clear from the data achieved that the above explanation, which is obtained from the survey of PLA, still applies to ABS material.

## 3. Analysis with Chromatology

**Figure 7:**
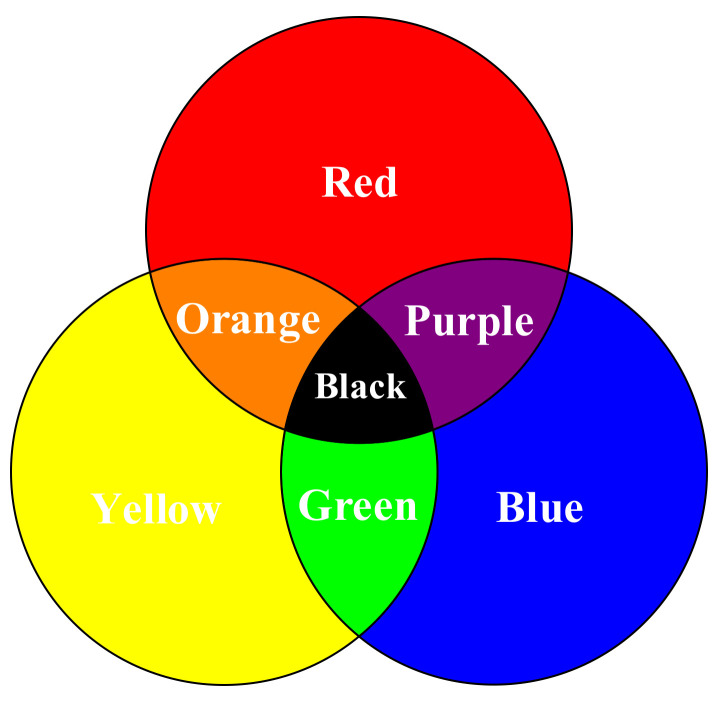
Color circle.

According to additive methods of color reproduction [26] or color-mixing theory [27], all the colors are produced by adding or blending together in different proportions of primary colors. Primary colors are three colors that are independent of each other, none of which can be produced by mixing the other two colors. In general, for pigments and links, red, yellow, and blue are the three primary colors [28]. As said before, the difference in material behaviors of various filament colors is due to the additives. Therefore, it is necessary to analyze whether the mechanical properties are also in line with the color-mixing theory. Of the selected colors, three colors (red, yellow, and blue) serve as the primary colors, while the other colors (orange, purple, green, and black) are just their mixtures, as presented in Figure 7. This is the reason that we select these colors as experimental objects.

The color-matching function can be written as [26]:

(1) M≡r(R)+y(Y)+b(B)

where *M* donates the mixed color; *R*, *Y*, and *B* donate three primary colors: red, yellow, and blue, respectively; and *r*, *y*, and *b* donate the corresponding non-negative mixing ratio. For example, when red and yellow are mixed in the ratio of *r*:*b* = 1:1, orange will be obtained. In the case of *r*:*b* ≠ 1:1, the mixture color may be reddish-orange, yellowish-orange, etc., depending on the appropriate adjustment of relative quantity.

### 3.1. Experiment with Two-Mixed Colors

In this section, we will investigate the mechanical strengths of two colors and their mixture to find the relationship between them. Take PLA colors: red, blue, and purple, as an example. According to the color circle in Figure 7, the mixture of red and blue will produce purple. Let *R^TS^*, *B^TS^*, and *P^TS^* donate the tensile strength of red, blue, and purple, respectively. If the mechanical properties of the material colors also conform to the color-mixing theory, the following equation will be obtained:

(2) $$P^{TS}=\lambda_{1}R^{TS}+\lambda_{2}B^{TS}$$

**Figure 8:**
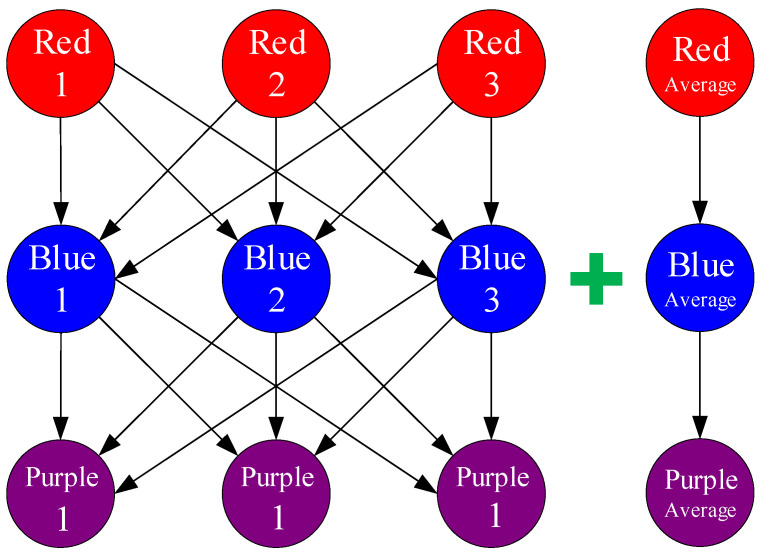
28 groups of permutation for Red + Blue → Purple.

The coefficients λ_1_ and λ_2_ can be interpreted as the ratio of tensile strength of red color and blue color, respectively, which can be theoretically calculated by solving the equation above. However, considering the influence of various errors, such as experimental error and printing error, these parameters can be obtained by using the least-squares method (LSM) to solve the mathematical problem as follows:

(3) $$Q=\min\sum_{i=1}^{n}{\left(\lambda_{1}R_{i}^{TS}+\lambda_{2}B_{i}^{TS}-P_{i}^{TS}\right)}^{2}$$

where *n* represents the number of experimental data. Since each colored sample has three specimens for the tensile test, there are in total 3 × 3 × 3 = 27 combinations of experimental data for three colors without repetition. In addition, including the group of the combination of average value, there are 28 groups in total, meaning *n* = 28, as shown in Figure 8.

The tensile strengths of all specimens for these three colors are tabulated in Table 3. By solving the extreme value of Formula (3) with 28 sub-equations through MATLAB, the parameters $\;\lambda_{1}^{TS}=0.44$, $\;\lambda_{2}^{TS}=0.62$ can be obtained. Then, the fitted (predicted) tensile strength for purple color is calculated as 52.22 MPa, whose relative error is 0.54% compared with actual (experimental) tensile strength (52.5 MPa). The result shows that the difference between the experimental value and the estimated value is very small.

**Figure 9:**
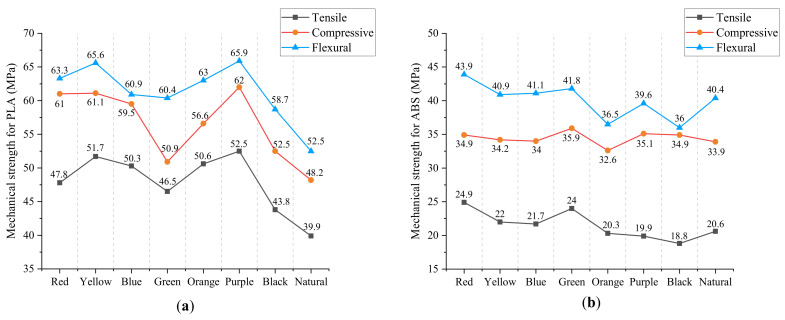
Mechanical strengths for different materials: (**a**) PLA and (**b**) ABS.

To better illustrate the internal relationship between different mechanical behaviors, the tensile strength, compressive strength, and flexural strength of PLA and ABS in various colors in Table 2 are plotted in Figure 9, respectively. Overall, flexural strength > compressive strength > tensile strength, both for PLA and ABS, which can be observed from the diagram. In addition, the three different mechanical strengths follow almost the same increasing and decreasing tendency, despite individual points. Consequently, tensile strength and flexural strength can be evaluated via compressive strength (Case 2), and tensile strength and compressive strength can be evaluated via flexural strength as well (Case 3). By observing the fitting values in Table 4, we can find that the relative fitting error can become larger or smaller when using ratio coefficients to evaluate other strengths. Even so, the maximum relative error for all mechanical strengths is less than 3.5%, proving the prediction is relatively accurate and satisfactory.

In addition, Table 4 shows the illustrative comparisons of parameters λ_1_ and λ_2_ obtained from different mechanical strengths. It is obvious to note that values are affected by the type of mechanical strength, due to varying degrees of data dispersion. Nevertheless, the difference has little effect on the predicted value, meaning the prediction method with color-matching theory is relatively robust and effective.

In order to verify the practicality of the prediction method for other situations, two groups of other color combinations for PLA are tested. On the other hand, to examine the difference between different materials, mechanical tests for ABS specimens of the same combination of colors are also carried out. Without loss of generality, the ratio coefficients are obtained by applying the LSM for different mechanical strengths, and the parameters in the situation where the maximum error emerges are presented.

The data in Table 5 reveal that the mechanical properties of other colors for PLA agree well with the color-mixing theory. The relative errors between actual values and predicted values are always within the range of 7%. Similarly, the theory also applies to ABS, whose relative error is no more than 7% in all cases, proving the generality and reasonability of the prediction method. Additionally, it can be seen from Table 6 that, compared with the results from Table 4, the parameters λ_1_ and λ_2_ obtained are obviously different for ABS and PLA, even in the same combination of colors. In the case of “Red + Blue→Purple”, the parameters obtained from flexural strength for PLA are $\;\lambda_{1}^{FS}=0.33$ and $\;\lambda_{2}^{FS}=0.72$; While these two parameters change to $\;\lambda_{1}^{FS}=0.14$ and $\;\lambda_{2}^{FS}=0.82$ for ABS in the same condition. Consequently, it is reasonable to demonstrate that the type of material has a significant effect on coefficient values.

### 3.2. Experiment with Three-Mixed Colors

In this subsection, the case where three colors are mixed is tested. According to the color circle in Figure 7, the mixture of red, blue, and yellow will produce black. Similarly, the Formula (2) will change as follows:

(4) $$Bk^{TS}=\lambda_{1}R^{TS}+\lambda_{2}B^{TS}+\lambda_{3}Y^{TS}$$

where *R^T^*, *B^T^*, *Y^T^*, and *Bk^T^* donate the tensile strength of red, blue, yellow and black, respectively. λ_1_, λ_2_, and λ_3_ represent the corresponding ratio coefficient for each primary color, which can be obtained by solving Formula (4):

(5) $$Q=\min\sum_{i=1}^{n}{\left(\lambda_{1}R_{i}^{TS}+\lambda_{2}B_{i}^{TS}+\lambda_{3}Y_{i}^{TS}-Bk_{i}^{TS}\right)}^{2}$$

Table 7 shows results of the PLA and ABS specimens with three-mixed colors. In a qualitative analysis, the predicted values match well with actual values, with the maximum relative error of about 6%. Comparatively, the prediction for PLA seems to be more precise than ABS. As discussed before, the flexural strengths of various colors for ABS are closer to each other, which leads to larger fitting errors by LSM. Overall, the color-mixing theory is applicable to predict the mechanical strength of three-mixed colors.

## 4. Confirmation Test

**Figure 10:**
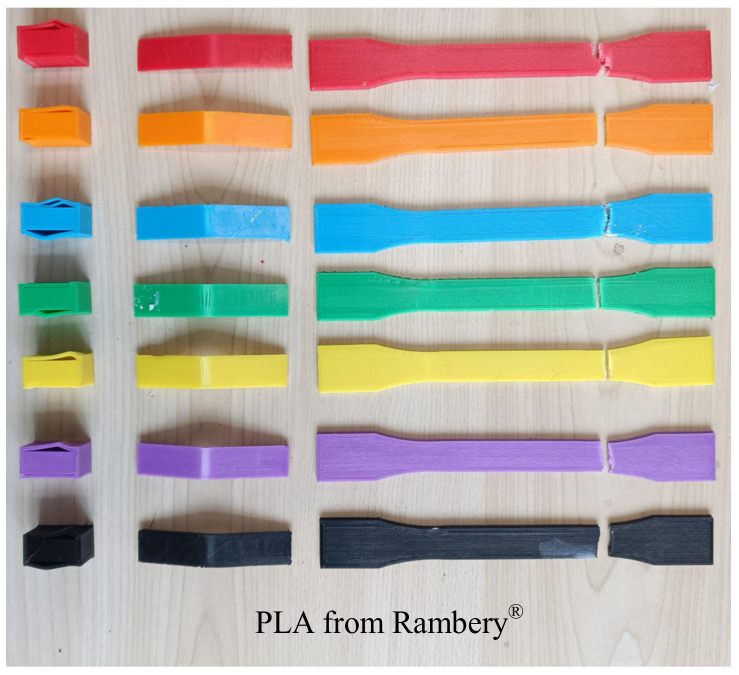
Broken PLA specimens from Rambery^®^ after confirmation test.

There is a large number of FDM material manufacturers available on the market. However, due to the lack of standards for AM, the same material from different manufacturers may exhibit significant discrepancy in property [29]. Therefore, it is necessary to check whether the conclusions obtained can be extended to other colored filaments from different suppliers. The PLA filaments used for the confirmation test were provided by Rambery^®^ (Cixi, China), as shown in Figure 10. In this time, seven colored filaments (red, yellow, blue, green, orange, purple and black) were selected to demonstrate the applicability of the approach. The average tensile strength, compressive strength and flexural strengths are calculated and tabulated in Table 8.

A close examination of Table 8 shows that compressive strength > flexural strength > tensile strength under the comprehensive consideration of all colors, which is different from PLA material from eSUN^®^. Moreover, even in the same color, the PLA material from other manufactures exhibit very different performance. For example, the mechanical strengths of the red sample from eSUN^®^ are 47.8 MPa for tension, 61 for compression and 63.3 for flexure, while the values become 37 MPa, 62.2 MPa and 44.1 MPa, respectively, for the sample from Rambery^®^ in the same color, demonstrating the importance of considering different performances caused by manufacturers in the selection of filaments [29].

**Figure 11:**
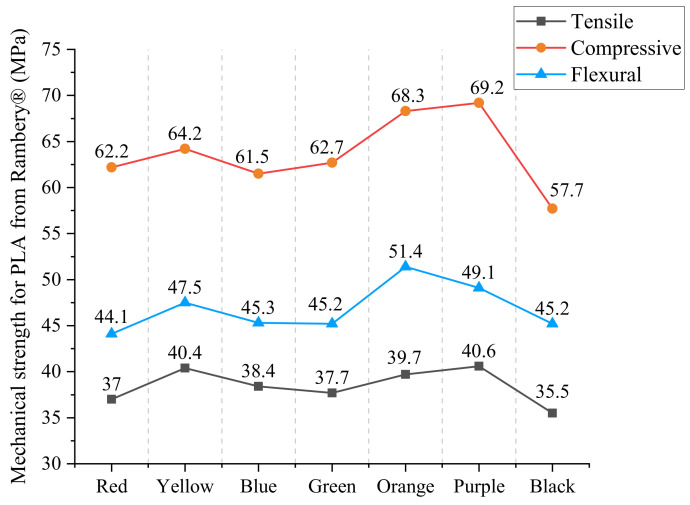
Mechanical strengths for PLA material from Rambery^®^.

In the interest of clarity, Figure 11 contains the plot of data of three mechanical strengths obtained via confirmation testing. As with Figure 9, the experimental result shows that strengths of Rambery^®^ samples printed in different colors also follow almost the same rising and falling curve in spite of individual points. That is, in the order of red→yellow→blue→green→orange→purple→black, the values first increase and decrease, and then increase and decrease again, meaning there is a qualitatively good agreement of strength trend between the materials from two suppliers.

Similarly, we use the LSM and color-mixing theory to evaluate the properties of mixed colors, just as discussed in the section before. Table 9 contains the numerical values leading to the maximum error of each group through different strength fitting. As can be seen, the maximum fitting error is within 5% in all cases (two-mixed colors and three-mixed colors), proving the applicability and precision of this approach.

## 5. Conclusions

This paper evaluated the influence of filament colors on mechanical characteristics of FDM products. The mechanical properties of ABS and PLA specimens printed using eight colors are characterized to determine tensile strength, compressive strength, and flexural strength. The analysis of experimental results presents that the color of the material plays a significant role in the performance of FDM products. Different filament colors affected the properties of the specimens to varying degrees. Samples chosen from the same eSUN^®^ PLA material but in various colors show the maximum difference of 29% in mechanical characteristics (tensile strength). On the one hand, the highest values for three kinds of mechanical strengths were all reached by the samples with purple color. On the other hand, the lowest mechanical strengths were reached by the samples with natural color. While for ABS, the maximum difference between different colors can be up to 36%, which also appears in tensile strength. In addition, the black samples achieved the lowest mechanical strength in all cases. On the contrary, the red samples achieved the highest tensile and compressive strength, while the green samples achieved the highest flexural strength. In summary, the influence of material color cannot be negligible, especially when studying the effect of process parameters on the structural performance of FDM parts.

At the same time, the achieved data show that the distribution trends of different mechanical strengths for different colors are consistent. That is to say, if a color has a higher (or lower) tensile strength when compared with other colors, its compressive strength and flexural strength are generally higher (or lower) as well. Furthermore, this study presents a novel approach for predicting mechanical strength with different colors using the color-mixing theory. By fitting the ratio coefficients using the LSM, the mechanical strength (tensile, compressive, and flexural) of mixed colors can be evaluated. The relative errors between the predicted value and actual value are always within the range of 7% for both PLA and ABS.

In addition, a confirmation test was performed on seven colored Rembery^®^ PLA material in an effort to verify the extensibility of this approach to filaments from different manufacturers. The experimental data indicates a good agreement of trend curves for three kinds of mechanical strengths from filaments of two brands. The statistical analysis indicates that the maximum fitting errors of strengths for all color mixture combinations are always within 5%, free from variances in filament manufacturer. The results presented clearly confirm the effectiveness and applicability of the prediction approach.

## 6. Discussion and Future Perspectives

This study reveals the relationship between mechanical characteristics and mixture of colors. Besides the complex parametric optimization approach [30,31], users can also improve strengths of their products by choosing proper colored filament, which is much easier to achieve. However, this preliminary research has several limitations and needs to be improved in the future:

First of all, the experimental materials are limited to ABS and PLA. Although previous studies have demonstrated that color additives to PLA material can lead to changes in mechanical properties, the degree of impact is different for different materials, as shown in this study. Therefore, it is necessary to further study the extent to which the color affects other FDM materials, such as nylon, polyetherimide, polycarbonates and others.

Meanwhile, this study used four sets of color mixtures to verify the color-mixing theory. According to color reproduction, the three primary colors can be mixed to produce almost all other colors. Therefore, more color combinations need to be tested in future work to verify the applicability of the theory.

Furthermore, a question that is not considered when processing data is the error of color. Due to the wide range of the color spectrum, it is difficult to tell whether a red color is “pure” red. For example, the mixture of red and yellow can be orange, reddish-orange or yellowish-orange. Apparently, the properties of these three colored filaments are not the same, which will affect the value of the fit coefficients λ_1_ and λ_1_. How to accurately describe the color of the filament is a challenge for both manufacturers and users.

At last, it has been proved that the color filler in filaments leads to discrepancy in mechanical performance. However, all filament manufacturers keep their respective colorant additives as proprietary trade secrets, making it challenging to explain the results as the additives are unknown. For this reason, a more detailed experiment before printing is needed to be conducted to obtain colored filaments through a filament production process, by blending a natural filament and color additive with known chemical characterization [32]. This would allow for understanding the relationship between color pigments and filament behaviors comprehensively and deeply, which is a research direction for future work.

## Figures and Tables

**Table 1:** Process parameters for FDM printing.

Material	Layer Thickness	Contour Width	Infill Density	Print Speed	Extrusion Temperature	Platform Temperature
PLA	0.15 mm	0.7 mm	100%	60 mm/s	200 °C	60 °C
ABS	0.15 mm	0.7 mm	100%	60 mm/s	250 °C	100 °C

**Table 2:** Average mechanical strengths of samples in different colors.

Color	PLA Mechanical Strength (MPa)			ABS Mechanical Strength (MPa)		
	Tensile	Compressive	Flexural	Tensile	Compressive	Flexural
Red	47.8	61.0	63.3	24.9	43.9	34.9
Yellow	51.7	61.1	65.6	22.0	40.9	34.2
Blue	50.3	59.5	60.9	21.7	41.1	34.0
Green	46.5	50.9	60.4	24.0	41.8	35.9
Orange	50.6	56.6	63.0	20.3	36.5	32.6
Purple	52.5	62.0	65.9	19.9	39.6	35.1
Black	43.8	52.5	58.7	18.8	36.0	32.5
Natural	39.9	48.2	52.5	20.6	40.4	33.9

**Table 3:** Tensile strength data of PLA specimens in red, blue and purple color.

Mechanical Strength (MPa)	Color	Red	Blue	Purple
Tensile	Specimen 1	47.9	49.9	52.6
	Specimen 2	47.3	50.6	52.9
	Specimen 3	48.1	50.4	51.9
	Average	47.8	50.3	52.5
Compressive	Specimen 1	60.9	59.4	61.8
	Specimen 2	61.3	59.6	62.1
	Specimen 3	60.9	59.7	62.0
	Average	61.0	59.5	62.0
Flexural	Specimen 1	62.1	61.1	65.9
	Specimen 2	64.4	59.9	65.8
	Specimen 3	63.5	61.7	65.9
	Average	63.3	60.9	65.9

**Table 4:** Predicated mechanical strengths for PLA samples in red, blue and purple color.

PLA	Case 1	Prediction with parameter $\;\lambda_{1}^{TS}=0.44$, $\lambda_{2}^{TS}=0.62\;$ from tensile strength					
		Average strength (MPa)	Red	Blue	Purple	Purple *	Error
		Tensile	47.8	50.3	52.5	52.22	0.54%
		Compressive	61.0	59.5	62.0	63.73	2.79%
		Flexural	63.3	60.9	65.9	65.61	0.44%
	Case 2	Prediction with parameters $\;\lambda_{1}^{CS}=0.32$, $\lambda_{2}^{CS}=0.71\;$ from compressive strength					
		Average strength (MPa)	Red	Blue	Purple	Purple *	Error
		Tensile	47.8	50.3	52.5	51.01	2.84%
		Compressive	61.0	59.5	62.0	61.77	0.38%
		Flexural	63.3	60.9	65.9	63.50	3.64%
	Case 3	Prediction with parameters $\;\lambda_{1}^{FS}=0.42$, $\lambda_{2}^{FS}=0.65$ from flexural strength					
		Average strength (MPa)	Red	Blue	Purple	Purple *	Error
		Tensile	47.8	50.3	52.5	52.77	0.52%
		Compressive	61.0	59.5	62.0	64.30	3.71%
		Flexural	63.3	60.9	65.9	66.17	0.41%

* represents the predicted value (the same below)

**Table 5:** Predicated values for mechanical strengths of PLA with two-mixed colors.

PLA	Group 1	Prediction with parameters $\;\lambda_{1}^{TS}=0.17$, $\lambda_{2}^{TS}=0.79$ from tensile strength					
		Average strength (MPa)	Red	Yellow	Orange	Orange *	Error
		Tensile	47.8	51.7	50.6	48.97	3.22%
		Compressive	61	61.1	56.6	58.64	3.60%
		Flexural	63.3	65.6	63	62.59	0.65%
	Group 2	Prediction with parameters $\lambda_{1}^{CS}=0.70$, $\lambda_{2}^{CS}=0.17$ from compressive strength					
		Average strength (MPa)	Yellow	Blue	Green	Green *	Error
		Tensile	51.7	50.3	46.5	44.74	3.78%
		Compressive	61.1	59.5	50.9	52.89	3.91%
		Flexural	65.6	60.9	60.4	56.27	6.84%

**Table 6:** Predicated values for mechanical strengths of ABS with two-mixed colors.

ABS	Group 1	Prediction with parameters $\lambda_{1}^{CS}=0.13$, $\lambda_{2}^{CS}=0.78$ from compressive strength					
		Average strength (MPa)	Red	Yellow	Orange	Orange *	Error
		Tensile	24.9	22	20.3	20.40	0.49%
		Compressive	43.9	40.9	36.5	37.61	3.04%
		Flexural	34.9	34.2	32.6	31.21	4.26%
	Group 2	Prediction with parameters $\lambda_{1}^{FS}=0.14$, $\lambda_{2}^{FS}=0.82$ from flexural strength					
		Average strength (MPa)	Red	Blue	Purple	Purple *	Error
		Tensile	24.9	21.7	19.9	21.28	6.93%
		Compressive	43.9	41.1	39.6	39.85	0.63%
		Flexural	34.9	34.0	35.1	32.77	6.65%
	Group 3	Prediction with parameters $\lambda_{1}^{CS}=0.85$, $\lambda_{2}^{CS}=0.20$ from compressive strength					
		Average strength (MPa)	Yellow	Blue	Green	Green*	Error
		Tensile	22.0	21.7	24.0	23.04	4.00%
		Compressive	40.9	41.1	41.8	42.99	2.85%
		Flexural	34.2	34.0	35.9	35.87	0.08%

**Table 7:** Predicated values for mechanical strengths with four colors.

PLA	Prediction with parameters $\;\lambda_{1}^{FS}=0.46$, $\lambda_{2}^{FS}=0.19$, $\lambda_{3}^{FS}=0.25$ from flexural strength						
	Average strength (MPa)	Red	Yellow	Blue	Black	Black *	Error
	Tensile	47.8	51.7	50.3	43.8	44.39	1.35%
	Compressive	61	61.1	59.5	52.5	54.54	3.89%
	Flexural	63.3	65.6	60.9	58.7	56.81	3.22%
ABS	Prediction with parameters $\lambda_{1}^{FS}=0.12$, $\lambda_{2}^{FS}=0.64$, $\lambda_{3}^{FS}=0.13$ from flexural strength						
	Average strength (MPa)	Red	Yellow	Blue	Black	Black *	Error
	Tensile	24.9	22	21.7	18.8	19.89	5.80%
	Compressive	43.9	40.9	41.1	36.0	36.79	2.19%
	Flexural	34.9	34.2	34.0	32.5	30.50	6.15%

**Table 8:** Average mechanical strengths of colored PLA samples from Rambery^®^.

Mechanical Strength (MPa)	Red	Yellow	Blue	Green	Orange	Purple	Black
Tensile	37.0	40.4	38.4	37.7	39.7	40.6	35.5
Compressive	62.2	64.2	61.5	62.7	68.3	69.2	57.7
Flexural	44.1	47.5	45.3	45.2	51.4	49.1	45.2

**Table 9:** Predicated values for mechanical strengths of PLA from Rambery^®^.

Group 1	Prediction with parameters $\;\lambda_{1}^{FS}=0.65$ $,\;\lambda_{2}^{FS}=0.46$ from flexural strength						
	Average strength (MPa)	Red	Blue	Purple	Purple *	Error	
	Tensile	37.0	38.4	40.6	41.71	2.73%	
	Compressive	62.2	61.5	69.2	68.72	0.69%	
	Flexural	44.1	45.3	49.1	49.50	0.81%	
Group 2	Prediction with parameters $\lambda_{1}^{CS}=0.40$, $\lambda_{2}^{CS}=0.66$ from compressive strength						
	Average strength (MPa)	Red	Yellow	Orange	Orange *	Error	
	Tensile	37.0	40.4	39.7	41.46	4.43%	
	Compressive	62.2	64.2	68.3	67.25	1.54%	
	Flexural	44.1	47.5	51.4	48.99	4.69%	
Group 3	Prediction with parameters $\;\lambda_{1}^{CS}=0.67$ $,\;\lambda_{2}^{CS}=0.31$ from compressive strength						
	Average strength (MPa)	Yellow	Blue	Green	Green *	Error	
	Tensile	40.4	38.4	37.7	38.97	3.37%	
	Compressive	64.2	61.5	62.7	62.08	0.99%	
	Flexural	47.5	45.3	45.2	45.87	1.48%	
Group 4	Prediction with parameters $\lambda_{1}^{TS}=0.4$, $\lambda_{2}^{TS}=0.38$ $\lambda_{3}^{TS}=0.17\;$ from tensile strength						
	Average strength (MPa)	Red	Yellow	Blue	Black	Black *	Error
	Tensile	37.0	40.4	38.4	35.5	36.68	3.32%
	Compressive	62.2	64.2	61.5	57.7	59.73	3.52%
	Flexural	44.1	47.5	45.3	45.2	43.39	4.00%

## Data Availability

Not applicable.
